# Supplementary material for: YeiE regulates YeiH to implement sulfite stress resistance in Salmonella enterica serotype Typhimurium
Source: J Bacteriol. 2026 Jan 12;208(2):e00431-25. doi: 10.1128/jb.00431-25 (PMC12880088; doi:10.1128/jb.00431-25)
Supplement: Figure S1 — Sequences of yeiE and yeiH promoters used in pCS26-Pac to drive luciferase expression. [file jb.00431-25-s0001.pdf]

Figure S1: Sequences of P_{yeiE} (A) and P_{yeiH} (B) used to drive luciferase expression in pCS26-Pac.

A.

```
5′ GTGTGCCTGTCCAGACCAAAGACTTTTTGGCCTAAGAAGCAGGCCAGCATGAACGTGCTG
3′ CACACGGACAGGTCTGGTTTCTGAAAAACCGGATTCTTCGTCCGGTCGTACTTGCACGAC   60

   GAAAGCGTTAATACGTCAATCACAATGCCGCTAATGCCCACGTCGGCGATTTGTGAAAAG
   CTTTCGCAATTATGCAGTTAGTGTTACGGCGATTACGGGTGCAGCCGCTAAACACTTTTC   120

   GTAAGGCGGAAGCCGTAAAGGATAATCCCTAAACGCAGCAGATGTTGTTTTGCAAACAGC
   CATTCCGCCTTCGGCATTTCCTATTAGGGATTTGCGTCGTCTACAACAAAACGTTTGTCG   180

   ACGCCGCCGTCGCATTGTTTCCATATTTGTGGATAGATGGTATTCCCGATAACCATACCC
   TGCGGCGGCAGCGTAACAAAGGTATAAACACCTATCTACCATAAGGGCTATTGGTATGGG   240

   AGCAGGATAGCCAGGGTCAGGGCGCTGAACCCCGCGCCTGCAACGGCAGGGATAGCGCCG
   TCGTCCTATCGGTCCCAGTCCCGCGACTTGGGGCGCGGACGTTGCCGTCCCTATCGCGGC   300

   CCCCACAGGGCGACTCCTGTAATGACGGCGCTCAGAGCGAGCCCCGGTATAAAATGCCAC
   GGGGTGTCCCGCTGAGGACATTACTGCCGCGAGTCTCGCTCGGGGCCATATTTTACGGTG   360

   ATTGTACGACGATGATTCTGCAAGGTGAGTTCTGTCATaaccttctcctgtatatggaca
   TAACATGCTGCTACTAAGACGTTCCACTCAAGACAGTAttggaagaggacatatacctgt   420
                                  <------
                                   yeiH

   ataaggttacggtgatctggtttaaaaataaaattgattatatatttataattaatcttt
   tattccaatgccactagaccaaatttttattttaactaatatataaatattaattagaaa   480

                                               yeiE
                                             ------>
   ataagtggtaagtgaaacgctatcggtgggatacgactATGCATATTACGCTACGACAAC
   tattcaccattcactttgcgatagccaccctatgctgaTACGTATAATGCGATGCTGTTG   540

   TTGAAGTGTTTGCTGAAGTACTGAAAAG    3′
   AACTTCACAAACGACTTCATGACTTTTC    5′  568
```

B.

```
5′ TTCCAGCCACGGCTCAGAGATAATCTCTGTACTGTGACATGGTCCTTCAATCAGGCCGAT
3′ AAGGTCGGTGCCGAGTCTCTATTAGAGACATGACACTGTACCAGGAAGTTAGTCCGGCTA   60

   ATCAACCCGAAAATCCAGCACCGCGTTAATCACGTCCTGGCTGTTTCCCACGCTGAGTTC
   TAGTTGGGCTTTTAGGTCGTGGCGCAATTAGTGCAGGACCGACAAAGGGTGCGACTCAAG   120

   CAGCGGCAAGGCCGGATAATGCTGGCGATAGCGGGCAATCATAGCGGGTAAAATGTAATT
   GTCGCCGTTCCGGCCTATTACGACCGCTATCGCCCGTTAGTATCGCCCATTTTACATTAA   180

   GCCGATGGTGCTACTGGCATAAACCCGTATCGCGCCGTTATCTTCACGAAATAGCTGCTC
   CGGCTACCACGATGACCGTATTTGGGCATAGCGCGGCAATAGAAGTGCTTTATCGACGAG   240

   AATTTCTATCGCTTGCTCCAGCAATGCCAGCGCGCGCGGGTATAACAAGCGCCCATGTTC
   TTAAAGATAGCGAACGAGGTCGTTACGGTCGCGCGCGCCCATATTGTTCGCGGGTACAAG   300

   GTTGACCACCAGCCGTTTCCCCACCCGATCAAACAACTGTACGCCGAGCTGACCTTCCAG
   CAACTGGTGGTCGGCAAAGGGGTGGGCTAGTTTGTTGACATGCGGCTCGACTGGAAGGTC   360

   ATCGGTGAGCGCGGCGCTAACGGCGGACTGCGATAATGACAGCATGACCGACGCCTGGGT
   TAGCCACTCGCGCCGCGATTGCCGCCTGACGCTATTACTGTCGTACTGGCTGCGGACCCA   420

   TGTTGAACCGCTTTTCAGTACTTCAGCAAACACTTCAAGTTGTCGTAGCGTAATATGCAT
   ACAACTTGGCGAAAAGTCATGAAGTCGTTTGTGAAGTTCAACAGCATCGCATTATACGTA   480
                                                        <-------
                                                          yeiE

   agtcgtatcccaccgatagcgtttcacttaccacttataaagattaattataaatatata
   tcagcatagggtggctatcgcaaagtgaatggtgaatatttctaattaatatttatatat   540

   atcaattttatttttaaaccagatcaccgtaaccttattgtccatatacaggagaaggtt
   tagttaaaataaaaatttggtctagtggcattggaataacaggtatatgtcctcttccaa   600
     yeiH
   ------>
   ATGACAGAACTCACCTTGCAGAATCATCGTCGTACAATGTGGCATTTTATACCGGGGCTC   3′
   TACTGTCTTGAGTGGAACGTCTTAGTAGCAGCATGTTACACCGTAAAATATGGCCCCGAG   5′  660
```
