# Supplementary figures and images for: YeiE regulates YeiH to implement sulfite stress resistance in Salmonella enterica serotype Typhimurium
Source: J Bacteriol. 2026 Jan 12;208(2):e00431-25. doi: 10.1128/jb.00431-25 (PMC12880088; doi:10.1128/jb.00431-25)

Figure S2: P<sub>yeiE</sub> DNA sequence used EMSA.

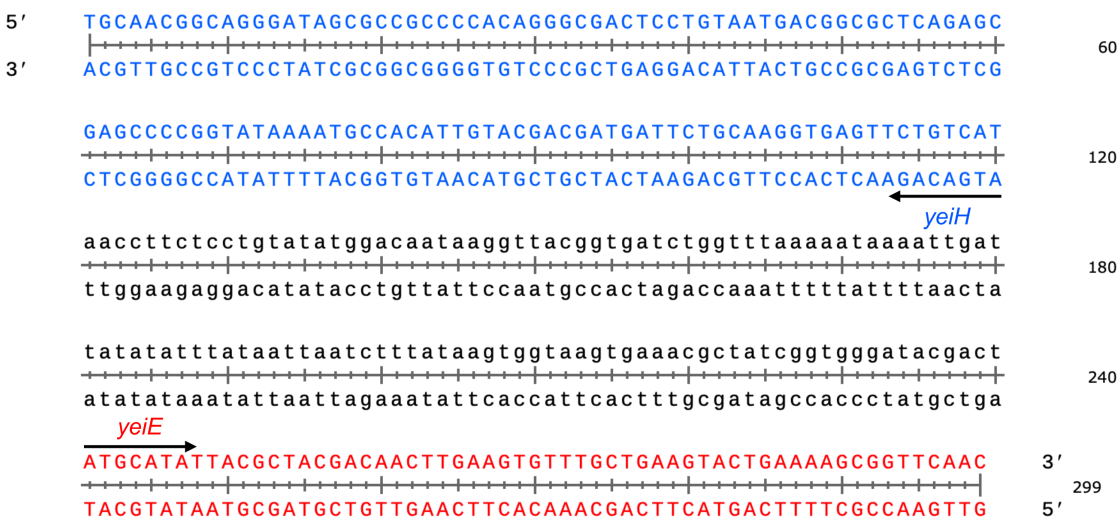

Supplement: Figure S2 — YeiE promoter sequence for EMSA. [file jb.00431-25-s0002.pdf]
